# Risk factors associated with changes in serum anti-Müllerian hormone levels before and after laparoscopic cystectomy for endometrioma

**DOI:** 10.3389/fendo.2024.1359649

**Published:** 2024-03-18

**Authors:** Chenyu Zhang, Xiaoyan Li, Yi Dai, Zhiyue Gu, Yushi Wu, Hailan Yan, Qiutong Li, Jinghua Shi, Jinhua Leng

**Affiliations:** ^1^Department of Obstetrics and Gynecology, Peking Union Medical College Hospital, Chinese Academy of Medical Sciences and Peking Union Medical College, Beijing, China; ^2^National Clinical Research Center for Obstetric & Gynecologic Diseases, Beijing, China

**Keywords:** endometrioma, laparoscopic cystectomy, AMH, ovarian reserve, ovarian cyst

## Abstract

**Background:**

The objective of our study was to investigate the risk factors for a decrease in ovarian reserve in patients with endometriomas after standardized laparoscopic procedures and evaluation to provide corresponding clinical guidance for patients with fertility requirements.

**Methods:**

Anti-Müllerian hormone (AMH) levels and other clinical data from 233 patients with endometriomas and 57 patients with non-endometrioma ovarian cysts admitted to the Peking Union Medical College Hospital between January 2018 and September 2023 were prospectively analysed. The pretreatment AMH levels of the study groups were compared to assess the impact of endometrioma on ovarian reserve, and the decrease in AMH after treatment was analysed to determine potential risk factors contributing to this change.

**Results:**

Pretreatment AMH levels did not significantly differ between patients with endometriomas and those with non-endometrioma ovarian cysts. Within the endometrioma group, older age, higher body mass index (BMI), and shorter menstrual cycles were found to be associated with decreased AMH levels prior to treatment (p<0.05). Participants presenting with bilateral cysts, advanced surgical staging, or a completely enclosed Douglas pouch demonstrated significantly lower levels of AMH prior to treatment compared to those without these conditions (p<0.05). Furthermore, their AMH levels further declined within one year after undergoing laparoscopic cystectomy (p<0.05). However, there was no difference in AMH levels after surgery between patients who successfully became pregnant and those who did not (p>0.05).

**Conclusion:**

Laparoscopic removal of endometriomas can adversely affect ovarian reserve, especially during bilateral cysts removal and when patients are diagnosed as having a higher stage of endometriosis, further impacting ovarian function. It should be noted that a decrease in AMH levels may not necessarily indicate an absolute decline in fertility. Therefore, it is crucial to conduct thorough patient evaluations and provide comprehensive patient education to offer appropriate guidance for fertility preservation.

## Background

Endometriosis (EM) is a prevalent gynaecological disorder in women and is defined by the ectopic implantation of endometrial cells outside the uterine lining. This condition can significantly compromise both the quality of life and reproductive health of affected individuals (1). Ovarian endometrioma (OEM) is the most common form of EM on the ovary and is known to diminish the ovarian reserve, making it a leading cause of EM-associated infertility. Although laparoscopic ovarian cystectomy used to be a primary therapeutic approach (2), there were challenges regarding the effectiveness of non-invasive treatments, such as dienogest (3), and their own effects on ovarian function and fertility; thus, the first-line surgical method and subsequent treatment to protect mainly fertility are the subject of ongoing debate.

Since there are no serum markers for direct quantification of the primordial follicle count, markers indicative of growing follicles have emerged as the optimal alternative for gauging ovarian reserve. For example, anti-Müllerian hormone (AMH) is secreted by granulosa cells in females and plays a pivotal role in inhibiting the activation of primordial follicles and the follicle-stimulating hormone (FSH)-driven growth of antral follicles, thus preventing undue follicular attrition. AMH levels closely correlate with the number of growing follicles (4), as these follicles release AMH, and AMH expression persists from secondary follicles to the antral follicle stage (5). Recently, AMH has gained traction in clinical practice as a reliable marker for ovarian reserve assessment because of its potential value in predicting the pregnancy outcome after laparoscopic excision of the endometrioma (6, 7). This is attributed to its consistent levels throughout the menstrual cycle, with minimal diurnal fluctuations (8) and high stability, making it a valuable tool for research.

Current studies present conflicting perspectives regarding the impact of endometriomas on ovarian reserve, potential influencing factors on AMH levels both before and after laparoscopic cystectomy, and the efficacy of AMH in predicting pregnancy outcomes. The heterogeneity of AMH measurements, surgeons, and treatment procedures poses a significant limitation in most relevant research studies. To address these limitations, we conducted a prospective cohort study of patients who underwent laparoscopic cystectomy performed by the same surgeon using consistent procedures. The main aim of our study was to examine controversial factors that might contribute to the decline in pre- and posttreatment AMH levels. We also investigated the difference in AMH concentration between endometriomas and non-endometrioma ovarian cysts and the association between AMH and fertility outcome. These findings will offer valuable insights for the accurate interpretation of pre- and posttreatment AMH values as well as for the prediction of compromised ovarian reserve after ovarian cystectomy in clinical practice.

## Methods

### Study protocol

In this investigation, our objective was to ascertain the viability of using the AMH concentration as an indicator of ovarian reserve function before and after surgical intervention and to examine the associated determinants. This was a prospective observational cohort study. The analysis of clinical data was conducted for patients who underwent laparoscopic ovarian cystectomy at Peking Union Medical College Hospital between January 2018 and September 2023 due to ovarian cyst diagnoses. The cohort included women of reproductive age, ranging from 18 to 45 years, who were diagnosed with an ovarian cyst but had not previously been subjected to invasive procedures such as aspiration, laparoscopy, or transabdominal cystectomy. All participants who underwent standardized laparoscopic cystectomy performed by the same senior surgeon, Dr. Leng, were included for postoperative comparison. The patients’ information, clinical characteristics, and surgical condition assessment were documented in a dedicated database specifically designed for this study. Based on histopathological findings, participants were categorized into an OEM (case) group and non-endometrioma (control) group. The control group comprised patients diagnosed with mature cystic teratomas, serous cystadenomas, mucinous cystadenomas, or borderline cystadenomas. The participants were requested to return to the outpatient department for a follow-up data collection at 1-month, 6-month, and 1-year intervals subsequent to the surgical procedure. The study protocol was approved by the Human Ethics Committee of Peking Union Medical College Hospital, and informed consent was obtained from all participants.

### Pathomorphology and biochemical analysis

The surgical tissue samples were promptly fixed in 10% neutral buffered formalin and sent to the Pathomorphology Department for preservation. Two pathomorphologists, who were unaware of the sample origins, independently evaluated the haematoxylin and eosin (H.E.) stained slides prepared from paraffin-embedded tissues, selecting appropriate samples for immunohistochemistry. Four-micron-thick sections were prepared for further analysis.

The AMH concentration and all blood tests were analysed at the Department of Clinical Laboratory, Peking Union Medical College Hospital. The AMH levels were simultaneously measured in all samples using the chemiluminescent kit ACCESS AMH on a UniCel® DxI 800 Immunoassay System (Beckman Coulter, Brea, CA, USA), with reference intervals ranging from 0.67 to 11.64 ng/ml.

### Outcomes

The dataset included preoperative data, including demographic details, such as age, body mass index (BMI), menstrual cycle length (MCL); clinical characteristics, such as disease duration, history of acute abdominal pain, records of prior abdominal surgeries excluding ovarian procedures (including appendectomy, caesarean section, renal surgeries, and gallbladder operations). The assessment of pain using the visual analogue scale (VAS) involves the utilization of a ruler equipped with precise markings, enabling patients to accurately indicate their pain intensity by marking the corresponding position on the ruler. Subsequently, physicians assign a score based on the marked position, facilitating a relatively objective evaluation of pain levels before and after treatment, while accounting for potential individual variations (9).

Laboratory test results, such as baseline AMH values, presurgical carbohydrate antigen 125(CA125) with reference intervals ranging from 0 to 35 U/ml, follicle-stimulating hormone(FSH)/luteinizing hormone (LH) ratios, haemoglobin with reference intervals ranging from 110 to 150g/l, and average postoperative AMH levels; and presurgical medication, including gonadotropin-releasing hormone agonist (GnRH-a) and oral contraceptives (OCs; drospirenone and ethinylestradiol tablets) were also analysed. The criteria for diagnosing adenomyosis are based on transvaginal ultrasound imaging, developed by the Morphological Uterus Sonographic Assessment (MUSA) Collaborative Group authorized by the International Federation of Gynaecology and Obstetrics (FIGO) in 2018 (10). Intraoperative circumstances, such as laterality of the cyst, maximum cyst dimension, cyst volume, surgical stage according to the revised American Fertility Society (rAFS) (11), deep infiltrating endometriosis (DIE) and adhesion of the Douglas pouch, were also recorded.

Postoperative follow-up data were extracted from outpatient medical records and telephone consultations. Given that age might influence the degree to which AMH levels decrease, AMH levels were recorded at specific intervals within the year following surgery. Postsurgical AMH levels were documented at 1-month, 6-month, and 1-year intervals. The average AMH concentration was utilized given its potential variability over time. All the data were gathered within a two-year window after surgery to account for age as a potential factor affecting AMH decline and postsurgical fertility outcomes in those reporting more than one year of presurgical infertility (the failure to achieve a pregnancy after 12 months or more of regular unprotected sexual intercourse) (12). Primary infertility is identified when a pregnancy has never been achieved by a person, and secondary infertility is when at least one prior pregnancy has been achieved. The sperm analysis of their spouses was conducted in accordance with the sixth edition of the World Health Organization (WHO) laboratory manual for the examination and processing of human semen (13). Additional data, such as postsurgical treatments and pregnancy outcomes for patients who reported infertility prior to the procedure, were also gathered.

### Statistical analysis

After conducting normality test on all continues variables, continuous variables were presented as the mean ± standard deviation (data exhibited a normal distribution) or medians (interquartile ranges, IQRs) (variables with skewed distributions). The group comparisons were conducted using Student’s t-test for continues variables with standard deviation, the Mann-Whitney U test or the Kruskal-Wallis test for two or more groups of independent samples with non-normal distribution. Categorical variables were presented as counts (percentages) and were compared using the chi-square test. The Spearman correlation analysis was employed to ascertain the association between continues variables and pretreatment levels and the decline of AMH levels. Linear regression models were used to adjust bias where appropriate. The data were processed and analysed using Statistical Program for the Social Sciences Statistics (SPSS, Version 22.0. Armonk, NY: IBM Corp). The statistical significance was determined when the p value < 0.05. The *post hoc* analysis was conducted to assess the statistical power of the study using G*Power (version 3.1.9.7 for Windows, Universität Düsseldorf).

## Results

In this study, a cohort of 290 patients was analysed. Of these, 233 patients were histologically confirmed to have an OEM following surgery, and 57 patients were diagnosed with a non-OEM. The *post hoc* analysis of the statistical powering was 0.96 analysed by G*Power (version 3.1.9.7 for Windows, Universität Düsseldorf). The demographic details are presented in **Supplementary Table 1**. There was no statistically significant difference in the demographic baseline of the participants across the groups.

### Clinical data of the compared participants

The median preoperative AMH concentrations were 2.76 (IQR: 3.38) ng/ml in the OEM group and 3.31 (IQR: 2.26) ng/ml in the control group, showing no significant difference between the two groups. The median disease duration for OEM patients was significantly longer than that for the non-OEM patients (p=0.010). Furthermore, patients diagnosed with OEM were more prone to experience severe dysmenorrhea with a higher VAS score (p<0.001), elevated CA125 levels (p<0.001), and history of acute abdominal pain (p=0.033). Additionally, coexistence with adenomyosis was more prevalent among OEM patients (p<0.001). Bilateral cysts were observed in 48.07% (112/233) of the patients with OEM, which was significantly higher than the 21.05% (12/57) in the patients with non-OEM, indicating a substantial disparity between the two groups (p<0.001). The maximum diameter of the unilateral cysts did not show a significant difference between the two groups (p=0.074). However, there was a noticeable disparity in the combined cyst volume between OEM patients and controls, possibly due to the lateral positioning of the cysts (p=0.029) (**Table 1**).

**Table 1:** Clinical data of the compared participants.

Characteristic	OEM N=233	Control N=57	P
AMH^1^ before surgery(ng/ml)	2.76(3.38)	3.31(2.26)	0.165^**^
FSH/LH^2^	1.49(1.27)	1.39(1.18)	0.655^**^
Disease duration (months)	12.00(31.50)	7.00(22.00)	0.010^**^
VAS score^3^	5.00(8.00)	0.00(4.00)	<0.001^**^
CA125(0-35 U/ml) ^4^	51.60(56.20)	16.40(9.25)	<0.001^**^
Acute abdominal pain			
No (n, %)	195(83.69)	54(94.74)	0.033^#^
Yes (n, %)	38(16.31)	3(5.26)	
Adenomyosis			
No (n, %)	166(71.24)	53(92.98)	<0.001^#^
Yes (n, %)	67(28.76)	4(7.02)	
Lateral			
Unilateral (n, %)	121(51.93)	45(78.95)	<0.001^#^
Bilateral (n, %)	112(48.07)	12(21.05)	
Cyst diameter(cm)	7.00(2.00)	6.00(3.00)	0.074^**^
Total cyst volume (cm^3^)	179.60(184.04)	113.10(202.63)	0.029^**^

^1^AMH, anti-Müllerian hormone.

^2^FSH, follicle-stimulating hormone; LH, luteinizing hormone.

^3^VAS, visual analogue scale.

^4^CA125, carbohydrate antigen 125.

^**^Mann-Whitney U test.

^#^chi-square test.

### Clinical characteristics and the decline of preoperative AMH level

To examine the factors influencing preoperative AMH levels, we conducted a Spearman correlation analysis on pretreatment AMH values for the two separate groups. Notably, age was significantly negatively correlated with AMH levels (r_EM=-0.462, p_EM<0.001; r_control=-0.418, p_control=0.001), while MCL was positively correlated with AMH levels (r_EM=0.213, p_EM=0.001; r_control=0.471, p_control<0.001). In the endometrioma group, a negative correlation was observed between BMI and AMH level (r_EM=-0.181, p_EM=0.006), but this correlation was not detected in the control group (r_control=-0.024, p_control=0.858) (**Table 2**). No correlations were detected among disease duration, VAS score, CA125 concentration, maximum cyst diameter, total cyst volume, and preoperative AMH level (**Supplementary Table 2**).

**Table 2:** Spearman Correlation Analysis between Clinical Characteristics and Preoperative AMH Level.

Category	OEM N=233		Control N=57	
	r_EM_	p_EM_	r_control_	p_control_
Age(yr)	-0.462	<0.001	-0.418	0.001
BMI^1^(kg/m^2^)	-0.181	0.006	-0.024	0.858
MCL^2^(month)	0.213	0.001	0.471	<0.001

^1^BMI, body mass index.

^2^MCL, menstrual cycle length.

In the OEM group, a comparative analysis was conducted on the preoperative AMH levels between patients with unilateral and bilateral cysts. The median AMH concentrations were 3.03 (IQR 3.61) ng/ml for those with a unilateral cyst and 2.71 (IQR 2.93) ng/ml for patients with bilateral cysts, demonstrating a statistically significant difference (p=0.041) (**Table 3**). Lateral disparities in AMH levels were also observed in the control group as well (p=0.048) (**Supplementary Table 3**). Additionally, patients with coexisting adenomyosis had a reduced AMH concentration, with a median of 2.45 (IQR 2.14) ng/ml, compared to those without adenomyosis, who had a median AMH concentration of 3.19 (IQR 3.32) ng/ml (p=0.004). After adjusting for age, no significant differences were observed between individuals with or without coexisting adenomyosis (**Table 3**).

**Table 3:** Differences in pretreatment AMH levels according to different clinical characteristics in the OEM cohort.

Category	OEM		
	N=233	AMH (ng/ml)	P_EM_
Lateral			
Unilateral	121	3.03(3.61)	0.041^**^
Bilateral	112	2.71(2.93)	
Adenomyosis			
No	166	3.19(3.32)	0.004^**^
Yes	67	2.45(2.14)	
Stage			
1	2	5.17(-)	0.008^a##^ 0.006^b**^
3	102	3.19(3.39)	
4	129	2.51(3.06)	
Douglas pouch			
Not closed	65	3.18(3.26)	0.006^c##^ 0.818^d**^ 0.013^e**^ 0.004^f**^
Semi-closed	72	3.40(3.90)	
Completely closed	96	2.48(2.84)	

^**^Mann-Whitney U test.

^##^Kruskal-Wallis test.

^a^The p value for the difference in pretreatment AMH concentration among different stages.

^b^The p value for the difference in pretreatment AMH concentration between patients with stage 3 and stage 4 disease.

^c^The p value for the difference in pretreatment AMH concentration among patients with different Douglas pouch conditions.

^d^The p value for the difference in pretreatment AMH concentration between patients with a non-closed or semi-closed Douglas pouch.

^e^The p value for the difference in pretreatment AMH concentration between patients with a non-closed or completely closed Douglas pouches.

^f^The p value for the difference in pretreatment AMH concentration between patients with semiclosed or completely closed Douglas pouches.

Furthermore, stage 4 patients had a lower median AMH level (2.51 (IQR 3.06) ng/ml) than did stage 3 patients, who had a median AMH level of 3.19 (IQR 3.39) ng/ml (p=0.006). Considering the state of the Douglas pouch, patients with a completely closed Douglas pouch had a significantly lower median AMH value of 2.48 (IQR 2.84) ng/ml than patients with a semiclosed pouch, with a median of 3.40 (IQR 3.90) ng/ml (p=0.013), or a nonclosed pouch, with a median of 3.18 (IQR 3.26) ng/ml (p=0.004). No significant differences were observed in AMH levels between patients with or without infertility complaints, or those with or without a previous history of acute abdominal pain or any other form of abdominal surgery (p>0.05) (**Supplementary Table 4**).

### Clinical characteristics and the decline of posttreatment AMH level

Follow-up information was available for one hundred and fourteen of the participants. The average AMH level for each individual within the year following surgery was determined. The median AMH concentration postoperatively was 1.12 (IQR 1.79) ng/ml, which significantly differed from the preoperative median AMH concentration of 3.03 (IQR 3.33) ng/ml (p<0.001). Given the baseline AMH discrepancies among patients, the postoperative AMH concentration was expressed as a percentage of the pretreatment AMH concentration to indicate the decrease in AMH concentration after surgery, with a median of 0.39 (IQR 0.40).

An analysis was performed to discern potential factors affecting the postsurgical reduction in AMH. However, factors such as age, BMI, length of menstrual cycle, disease duration, VAS score, CA125 concentration, pretreatment AMH concentration, total cyst volume, and maximum diameter of the cyst did not significantly correlate with the decrease in postoperative AMH concentration, as determined by the Spearman correlation test (p>0.05) (**Supplementary Table 5**).

More than 56% of patients (64/114) who underwent bilateral cystectomy experienced a more pronounced reduction in AMH levels than did those who underwent unilateral cystectomy (p<0.001). Patients with concurrent adenomyosis also had a more pronounced decrease in AMH levels than did those without adenomyosis (p=0.034). However, after adjusting for age, there was no statistically significant difference observed between patients with coexisting adenomyosis and those without (**Table 4**).

**Table 4:** Regressive postoperative AMH levels in patients with OEM across diverse clinical profiles.

Category	N=114	AMH%^1^	P
Lateral			
Unilateral	50(43.86)	0.59(0.40)	<0.001^**^
Bilateral	64(56.14)	0.26(0.28)	
Adenomyosis			
No	86(75.44)	0.45(0.42)	0.034^**^
Yes	28(24.56)	0.26(0.30)	
Stage			
1	1(0.88)	0.54	<0.001^a##^ <0.001^b**^
3	49(42.98)	0.55 (0.40)	
4	64(56.14)	0.29(0.35)	
Douglas pouch			
Non-closed	28(24.56)	0.54(0.39)	0.004^c##^ 0.444^d**^ 0.003^e**^ 0.013^f**^
Semi-closed	38(33.33)	0.46(0.43)	
Completely closed	48(42.11)	0.27(0.35)	

^**^Mann-Whitney U test.

^##^Kruskal-Wallis test.

^1^AMH%= posttreatment AMH/pretreatment AMH.

^a^The p value for the difference in AMH% among different stages.

^b^The p value for the difference in AMH% between patients with stage 3 and stage 4 disease.

^c^The p value for the difference in AMH% among patients with different Douglas pouch conditions.

^d^The p value for the difference in AMH% between patients with non-closed or semi-closed Douglas pouch.

^e^The p value for the difference in AMH% between patients with nonclosed or completely closed Douglas pouches.

^f^The p value for the difference in AMH% between patients with semiclosed or completely closed Douglas pouches.

Per the rAFS staging criteria, patients with stage 4 EM had steeper reductions in AMH levels than did those with stage 3 EM (p<0.001). Regarding the status of the Douglas pouch, patients with a completely closed Douglas pouch had a significantly lower AMH concentration than did those with either a non-closed (p=0.003) or semiclosed Douglas pouch (p=0.013) (**Table 4**).

Factors such as a history of OEM rupture, prior abdominal surgery, or the use of haemostatic or antiadhesive materials during surgery, the incidence of monolocular or multilocular cysts, treated with GnRH-a all did not impact the postsurgical decrease in AMH (p>0.05) (**Supplementary Table 6**).

### Pregnant outcomes of the infertility participants

Prior to surgery, a total of twenty-one participants reported infertility. Out of the participants who encountered difficulties in conceiving, 66.7% (14/21) experienced primary infertility, while 33.3% (7/21) faced secondary infertility. Among those with secondary infertility, one individual had previously undergone a successful full-term pregnancy. Approximately eighty-five percent (18/21) of their partners exhibited normal results in sperm examinations, while in 14.3% cases (3/21), the spouses displayed reduced sperm motility and were concurrently undergoing treatment while attempting conception. In all cases included in this study, the selected method of assisted reproduction was *in vitro* fertilization-embryo transfer (IVF-ET). Of the twenty-one patients who reported infertility prior to surgery, only one underwent surgery within a year, while the remaining twenty underwent surgery after more than a year. Of these twenty patients, thirty percent (6/20) achieved natural or artificially assisted conception and went on to have full-term deliveries. The postoperative AMH concentrations were 1.04 (IQR 0.89) ng/ml for patients with infertility and 0.31 (IQR 3.14) ng/ml for patients who became pregnant postoperatively, and the difference was not significant (p=0.885).

## Discussion

The prospective analysis of AMH levels prior to treatment in patients with OEM and other ovarian cysts suggests that OEM have no significant impact on pretreatment AMH levels compared to the control group. In terms of potential clinical factors affecting the pretreatment AMH levels, age and BMI were negatively correlated with AMH, while menstrual cycle length was positively correlated with AMH. Patients presenting with bilateral cysts, advanced-stage EM, or complete occlusion of the Douglas pouch had reduced levels of AMH prior to treatment, which further declined following treatment.

Consistent with Streuli’s findings (14), our study revealed no significant difference in preoperative AMH levels between OEM patients and their non-OEM counterparts when controlling for age and BMI. However, several studies have reported lower preoperative AMH levels and antral follicle counts (AFCs) in OEM patients than in their non-OEM counterparts (15–17). Several molecular investigations suggest that the peritoneal fluid of EM patients contains more proinflammatory, chemotactic, angiogenic, and oxidative stress factors, which may adversely affect ovarian function (18, 19).

The ovarian reserve, a function of the number and health of primordial follicles, decreases with age (20). We noted that older patients had reduced AMH levels, although age did not correlate with a more substantial decrease in postsurgical AMH. However, the relationship between AMH and BMI in reproductive-aged women is ambiguous. In our OEM cohort, BMI was inversely correlated with AMH; this association was not detected in the non-OEM group. A review posited an uncertain relationship between BMI and AMH in women without PCOS but a potential negative correlation in PCOS patients (21). The length of the menstrual cycle was also found to be positively correlated with pretreatment AMH levels, which is consistent with Younis’ research (22). A decrease in the number and quality of follicles in the ovary might result in a shortened follicular phase prior to ovulation, which manifests as a reduced MCL compared to that of normal individuals, regardless of age.

There is a significant decrease in postsurgical AMH levels, whether measured at 1, 6, or 12 months, compared to pretreatment AMH levels. Existing studies support the notion that patients who undergo laparoscopic ovarian cystectomy, regardless of whether it is for OEM or other benign cysts, experience a substantial decrease in postoperative AMH levels, indicating a diminished ovarian reserve (23, 24). The reason for this decrease could be accidental damage to healthy ovarian tissue (25), compromised ovarian blood flow postadhesiolysis (26), or thermal damage during coagulation of small bleeding vessels within the ovarian parenchyma (27). This decline might persist (28) or be transient (26, 29).

The pretreatment AMH level are significantly lower in cases of bilateral ovarian cysts compared to unilateral, and the posttreatment decline in AMH is more pronounced (30, 31). Evidence suggests that bilateral laparoscopic cystectomy increases the risk of premature ovarian failure and early menopause (32), potentially due to prolonged ovarian ischemia resulting from bilateral surgeries. Some studies note that cystectomy may remove portions of ovarian tissue during stripping of the OEM wall, thereby decreasing the follicle count (25). However, pathological results did not reveal a follicular morphology that is usually visible in normal ovarian tissue (33, 34). Notably, cyst size did not correlate with AMH reductions, consistent with findings from Somigliana (35) and Hirokawa (36).

The severity of EM and presence of DIE might have an impact on pre- and posttreatment AMH levels. Our research revealed that patients with advanced EM or a completely occluded Douglas pouch, indicative of severe pelvic adhesions due to EM, had reduced preoperative AMH levels and a more considerable postsurgical decline. The decrease in AMH pretreatment might be caused by chronic inflammation and increased levels of cytokines, especially IL-6, which have been shown to be associated with the occurrence of EM-associated infertility (37, 38). However, the enduring decrease after treatment may arise from compromised ovarian blood flow postadhesiolysis (26). A more advanced adhesion may lead to an increased vascular damage and a decreased postsurgical blood supply. The excision of DIE lesions has been reported to contribute to the improvement of the pregnancy rate as well (6). However, factors such as prior nongynecological abdominal surgeries or cyst ruptures did not affect AMH levels.

The haemostatic method used during surgery might affect postoperative AMH levels. While some studies favour suture haemostasis over bipolar coagulation for preserving ovarian function (26, 39–41), the protective effect of sutures on ovarian function may be limited (42, 43). A comparison between sutures and electric coagulation was not possible in this study due to the consistent use of electric coagulation for controlling bleeding at specific points during surgical procedures. However, we found no significant differences in AMH levels in our comparison of the use of haemostatic or antiadhesive materials during surgery.

Patients with adenomyosis had lower pretreatment AMH levels and a greater posttreatment decline, but their median age was higher than that of patients without adenomyosis, suggesting potential age-related bias. After adjusting for age, no significant differences were observed between individuals with or without coexisting adenomyosis. The relationship between adenomyosis and AMH concentration remains uncertain (44).

Our data did not reveal substantial impacts of GnRH-a or other drug treatments on ovarian reserve. The literature suggests variable AMH responses to GnRH-a, with some studies noting a transient AMH decline that eventually normalizes (45, 46). According to the 2022 guidelines of the European Society of Human Reproduction and Embryology (ESHRE) (47), it has been suggested that suppressing ovarian function does not yield improved fertility outcomes in women diagnosed with EM.

There are many factors that might influence the AMH concentration before or after treatment, and the main aim of all the studies was to investigate the ultimate effect on fertility. However, a diminished ovarian reserve seems not to be associated with reduced future reproductive capacity (7, 48). Of our sample, 20 patients reported infertility before surgery. The postoperative AMH concentrations were 1.04 (IQR 0.89) ng/ml for patients with infertility and 0.31 (IQR 3.14) ng/ml for patients who became pregnant postoperatively, while the difference was not significant (p=0.885). It is important to note that the limited sample size may have contributed to these non-significant findings. Despite women who have previously undergone laparoscopic cystectomy for OEM experiencing a lower number of retrieved oocytes and a reduced cumulative chance of achieving a live birth from a single IVF cycle, compared to those with other types of infertility (49), there was no evidence suggesting any decline in oocyte (50) or embryo quality (51). Therefore, the rates of pregnancy and delivery remained fully comparable and overlap with those observed in cases of other causes of infertility (52, 53).

In order to provide the opportunity for superior quality stored oocytes and affording individuals additional time to contemplate their reproductive aspirations, oocyte cryopreservation has recently emerged as a promising therapeutic approach for patients diagnosed with OEM (54). The issue of whether pre-surgical cryopreservation of oocytes is advisable continues to be a subject of intense debate, requiring comprehensive assessment of patient-specific factors including potential complications, oocyte viability restoration, and cost-effectiveness (55, 56).

The strength of this study lies in its consistency: all patients underwent surgery performed by the same team using standardized procedures. This approach effectively reduces variability resulting from differences in surgical proficiency or other external factors. Additionally, we conducted a comprehensive analysis and evaluation of all potential factors present in clinics, regardless of their positive or negative results, to investigate their potential impact on pretreatment or posttreatment ovarian reserve. This research aims to assist clinicians in accurately assessing the severity of these influences and making informed clinical decisions. The limitations of individualized postoperative treatment regimens in real-world research underscore the imperative for further investigation through meticulously designed and executed prospective studies.

## Conclusion

In conclusion, laparoscopic cystectomy has a significant impact on ovarian reserve, particularly in patients who undergo bilateral cyst removal or have more severe endometriosis, rather than the presence of endometrioma itself. However, a decrease in AMH levels may not necessarily indicate an absolute decline in fertility outcomes. Therefore, it is crucial to thoroughly assess all factors that can influence ovarian reserve prior to surgery and ensure comprehensive patient education. Further research is needed to establish a stronger correlation between AMH levels and fertility outcomes.

## Data availability statement

The original contributions presented in the study are included in the article/**Supplementary Material**. Further inquiries can be directed to the corresponding authors.

## Ethics statement

The studies involving humans were approved by the Human Ethics Committee of Peking Union Medical College Hospital. The studies were conducted in accordance with the local legislation and institutional requirements. The participants provided their written informed consent to participate in this study. All participants provided informed consent for participation.

## Author contributions

CZ: Conceptualization, Data curation, Formal analysis, Funding acquisition, Investigation, Methodology, Project administration, Resources, Software, Supervision, Validation, Visualization, Writing – original draft, Writing – review & editing. XL: Writing – original draft, Writing – review & editing. YD: Conceptualization, Data curation, Formal analysis, Funding acquisition, Investigation, Methodology, Project administration, Resources, Software, Supervision, Validation, Visualization, Writing – original draft, Writing – review & editing. ZG: Conceptualization, Data curation, Formal analysis, Funding acquisition, Investigation, Methodology, Project administration, Resources, Software, Supervision, Validation, Visualization, Writing – original draft, Writing – review & editing. YW: Conceptualization, Data curation, Formal analysis, Funding acquisition, Investigation, Methodology, Project administration, Resources, Software, Supervision, Validation, Visualization, Writing – original draft, Writing – review & editing. HY: Conceptualization, Data curation, Formal analysis, Funding acquisition, Investigation, Methodology, Project administration, Resources, Software, Supervision, Validation, Visualization, Writing – original draft, Writing – review & editing. QL: Conceptualization, Data curation, Formal analysis, Funding acquisition, Investigation, Methodology, Project administration, Resources, Software, Supervision, Validation, Visualization, Writing – original draft, Writing – review & editing. JS: Conceptualization, Data curation, Formal analysis, Funding acquisition, Investigation, Methodology, Project administration, Resources, Software, Supervision, Validation, Visualization, Writing – original draft, Writing – review & editing. JL: Conceptualization, Data curation, Formal analysis, Funding acquisition, Investigation, Methodology, Project administration, Resources, Software, Supervision, Validation, Visualization, Writing – original draft, Writing – review & editing.
