# Supplementary material for: Risk factors associated with changes in serum anti-Müllerian hormone levels before and after laparoscopic cystectomy for endometrioma
Source: Front Endocrinol (Lausanne). 2024 Mar 18;15:1359649. doi: 10.3389/fendo.2024.1359649 (PMC10982650; doi:10.3389/fendo.2024.1359649)
Supplement: Supplementary file 1 [file Table_1.docx]

**Supplemental Table 1 Demographic data of the compared participants**

| Characteristic | OEM N=233 | Control N=57 | P |
| --- | --- | --- | --- |
| Age(yrs) | 31.97±5.37 | 30.56±5.73 | 0.081^*^ |
| BMI^1^ (kg/m^2)^ | 21.31±2.96 | 21.51±3.44 | 0.671^*^ |
| MCL^2^ (d) | 29.00(2.00) | 30.00(2.00) | 0.054^**^ |
| Haemoglobin (110-150g/l) | 128.00(15.00) | 126.00(10.00) | 0.424^**^ |
| Gravidity | | | |
| 0(n, %) | 157 (67.38) | 34 (59.65) | 0.270^#^ |
| ≥1(n, %) | 76 (32.62) | 23(40.35) |  |
| Parity | | | |
| 0(n, %) | 182(78.11) | 38(66.67) | 0.070^#^ |
| ≥1(n, %) | 51(21.89) | 19(33.33) |  |
| Infertility | | | |
| 0(n, %) | 202(86.70) | 54(94.74) | 0.109^#^ |
| ≥1(n, %) | 31(13.30) | 3(5.26) |  |
| History of abdominal surgery | | | |
| 0(n, %) | 192(82.40) | 46(80.70) | 0.764^#^ |
| ≥1(n, %) | 41(17.60) | 11(19.30) |  |

^1 BMI: body mass index^

^2 MCL: menstrual cycle length^

^* Student's t-test^

^** Mann-Whitney U test^

^# chi-square test^

**Supplemental Table 2 Spearman Correlation Analysis between Clinical Characteristics and Preoperative AMH Level**

| Category | OEM N=233 | | Control N=57 | |
| --- | --- | --- | --- | --- |
|  | r_EM_ | P_EM_ | r_control_ | P_control_ |
| Disease duration(month) | -0.073 | 0.270 | -0.196 | 0.145 |
| VAS^1^ | -0.054 | 0.415 | 0.026 | 0.849 |
| CA125^2^(0-35 U/ml) | <0.001 | 0.998 | 0.172 | 0.201 |
| Cyst max diameter(cm) | 0.005 | 0.936 | -0.084 | 0.534 |
| Total cyst volume(cm^3^) | <0.001 | 0.996 | -0.116 | 0.391 |

^1 VAS: visual analogue scale^

^2 CA125:^ ^carbohydrate antigen 125^

**Supplemental Table 3 Differences in Preoperative AMH Levels in Control Group According to Clinical Characteristics**

| Category | Control | | |
| --- | --- | --- | --- |
|  | N=57 | AMH (ng/ml) | P |
| Lateral |  |  |  |
| Unilateral | 45 | 3.61(2.77) | 0.048^**^ |
| Bilateral | 12 | 2.63(1.61) |  |
| Infertility |  |  |  |
| No | 54 | 3.26(2.44) | 0.943^**^ |
| Yes | 3 | 3.38(-) |  |
| Acute abdominal pain | |  |  |
| No | 54 | 3.26(2.26) | 0.163^**^ |
| Yes | 3 | 5.36(-) |  |
| History of abdominal surgery | |  |  |
| No | 46 | 3.00(2.26) | 0.217^**^ |
| Yes | 11 | 4.27(2.84) |  |
| Adenomyosis |  |  |  |
| No | 53 | 3.20(2.36) | 0.248^**^ |
| Yes | 4 | 4.36(1.72) |  |

^** Mann-Whitney U test^

**Supplemental Table 4 Differences in pretreatment AMH levels among different clinical characteristics in the OEM group**

| Category | OEM N=233 | | |
| --- | --- | --- | --- |
|  | N | AMH (ng/ml) | P |
| DIE^1^ |  |  | 0.222^**^ |
| No | 103 | 3.18(3.35) |  |
| Yes | 130 | 2.67(3.36) |  |
| Infertility |  |  |  |
| No | 202 | 2.94(3.36) | 0.212^**^ |
| Yes | 31 | 2.70(2.51) |  |
| Acute abdominal pain |  |  |  |
| No | 195 | 2.74(3.34) | 0.524^**^ |
| Yes | 38 | 3.10(3.61) |  |
| History of abdominal surgery |  |  |  |
| No | 192 | 2.98(3.34) | 0.101^**^ |
| Yes | 41 | 2.50(2.77) |  |

^1^ ^DIE: deep infiltrating endometriosis^

^** Mann-Whitney U test^

**Supplemental Table 5 Spearman Correlation Analysis between Clinical Characteristics and Decreases in Postoperative AMH Levels**

|  | Age | BMI^1^ | MCL^2^ | Disease duration | VAS^3^ | CA125^4^ | Preoperative AMH | Total cyst volume (cm^3^) | Cyst diameter(cm) |
| --- | --- | --- | --- | --- | --- | --- | --- | --- | --- |
| r | -0.132 | -0.094 | -0.125 | -0.098 | -0.012 | -0.145 | 0.036 | -0.129 | 0.159 |
| p | 0.162 | 0.318 | 0.185 | 0.298 | 0.901 | 0.124 | 0.705 | 0.170 | 0.269 |

^1 BMI: body mass index^

^2 MCL: menstrual cycle length^

^3 VAS: visual analogue scale^

^4 CA125: carbohydrate antigen 125^

**Supplemental Table 6 Decline in Postoperative AMH in OEM across Diverse Clinical Profiles**

| Category | N=114 (n, %) | AMH%^1^ | P |
| --- | --- | --- | --- |
| Acute abdominal pain |  |  |  |
| No | 91(79.82) | 0.36(0.42) | 0.818^**^ |
| Yes | 23(20.18) | 0.45(0.45) |  |
| History of abdominal surgery |  |  |  |
| No | 101(88.60) | 0.40(0.42) | 0.250^**^ |
| Yes | 13(11.40) | 0.28(0.42) |  |
| DIE^2^ |  |  |  |
| No | 39(34.21) | 0.44(0.44) | 0.685^**^ |
| Yes | 75(65.79) | 0.35(0.39) |  |
| Number of cyst chambers (unilateral N=50) | |  |  |
| monolocular | 8(16.00) | 0.76(0.41) | 0.101^**^ |
| multilocular | 42(84) | 0.56(0.45) |  |
| Hemostatic materials during the operation | |  |  |
| No | 14(12.28) | 0.53(0.57) | 0.225^**^ |
| Yes | 100(87.71) | 0.37(0.39) |  |
| Anti adhesive material during the operation | |  |  |
| No | 6(5.26) | 0.78(0.99) | 0.103^**^ |
| Yes | 108(94.74) | 0.37(0.39) |  |
| Drug therapy before surgery |  |  |  |
| None | 78(68.42) | 0.45(0.400) | 0.172^a##^  0.317^b**^ |
| GnRH-a^3^ | 35(30.70) | 0.32(0.43) |  |
| OCs^4^ | 1(0.88) | 1.17 |  |
| Drug therapy during whole process | |  |  |
| None | 13(11.40) | 0.18(0.55) | 0.261^c##^  0.147^d**^  0.183^e**^  0.499^f**^ |
| GnRH-a^3^ | 84(73.68) | 0.35(0.43) |  |
| OCs^4^ | 17(14.91) | 0.46(0.31) |  |

^1 AMH%= posttreatment AMH/pretreatment AMH.^

^2^ ^DIE: deep infiltrating endometriosis^

^3 GnRH-a: gonadotropin-releasing hormone agonist^

^4 OCs: oral contraceptives^

^** Mann-Whitney U test^

^## Kruskal-Wallis test^

^a The p value for the difference in AMH% among patients receiving different drug therapies before surgery^

^b The p value for the difference in AMH% between patients treated with GnRH-a and patients not receiving medical treatment before surgery^

^c The p value for the difference in AMH% among patients receiving different drug therapies during the whole process^

^d The p value for the difference in AMH% between patients treated with GnRH-a and patients not receiving medical treatment during the whole process^

^e The p value for the difference in AMH% between patients treated with OCs and patients not receiving medical treatment during the whole process^

^f The p value for the difference in AMH% between patients treated with GnRH-a and those treated with OCs during the whole process^
